# Supplementary material for: Predicting thickness perception of liquid food products from their non-Newtonian rheology
Source: Nat Commun. 2021 Nov 3;12:6328. doi: 10.1038/s41467-021-26687-w (PMC8566491; doi:10.1038/s41467-021-26687-w)
Supplement: Supplementary file 1 — Supplementary Information [file 41467_2021_26687_MOESM1_ESM.pdf]

Supplementary Information for: Predicting thickness perception of liquid food products from their non-Newtonian rheology

Antoine Deblais,[1,2,*] Elyn den Hollander,[1] Claire Boucon,[1] Annelies E. Blok,[3] Bastiaan Veltkamp,[2] Panayiotis Voudouris,[1] Peter Versluis,[1] Hyun-Jung Kim,[1] Michel Mellema,[1] Markus Stieger,[3,4] Daniel Bonn,[2] and Krassimir P. Velikov[1,2,5,†]

[1] *Unilever Innovation Centre Wageningen, Bronland 14, 6708 WH Wageningen, The Netherlands.*
[2] *Van der Waals-Zeeman Institute, IoP, University of Amsterdam, Science Park 904, 1098 XH Amsterdam, Netherlands.*
[3] *Food Quality and Design, Wageningen University, P.O. Box 17, 6700 AA Wageningen, The Netherlands.*
[4] *Division of Human Nutrition and Health, Wageningen University, P.O. Box 17, 6700 AA Wageningen, The Netherlands.*
[5] *Soft Condensed Matter, Debye Institute for NanoMaterials Science, Utrecht University, Princetonplein 5, 3584 CC, Utrecht, The Netherlands.*

(Dated: October 18, 2021)

Supplementary Note 1: Viscoelasticy of the liquid samples

We investigate bouillon samples with different compositions. The presence of xanthan gum and starch eventually contributes to weak viscoelasticity appearing in the samples as has been shown in [1]. This may cause an additional contribution to the flow. We checked the eventual viscoelastic response of our materials by performing classical filament rheometry measurements [2]. The evolution of the minimum neck diameter $D_{min}(t)$ with time is shown in Figure 1 for the two sets of bouillons (and normalized by the initial neck diameter D_0). From these results, we conclude that few samples show (very) weak-viscoelastic behavior by exhibiting a very short characteristic exponential thinning decay regime $D_{\mathrm{min}}/D_0 = \exp(-t/3\lambda_0)$ (solid lines in Fig. 1). In Table we list the maximum relaxation times λ_0 obtained from fitting these experiments. The longest time is $\lambda_0 = 7$ ms. Overall, the relaxation times measured here in all samples are very small compared to the relaxation times for materials considered in the work of G. Brindley *et al.* [3], that shows that elasticity does not play a role in squeezing flows for such low elasticity. Considering also that only low forces ($F_N = 500$ mN) and long time scales are involved in our dynamic squeezing, no effect of elasticity is expected for our solutions.

Supplementary Note 2: Agreement with the Cox-Merz relationship

We performed rheology measurements of $G^{'}$ and $G^{''}$ as a function of frequency for the sample with the highest concentration of xanthan gum (*i.e.*, sample 14). Strain sweep experiments indicate that the linear viscoelastic regime for the sample extends to a strain $\gamma \leq 0.5\%$. We thus performed frequency sweep measurements at $\gamma_0 = 0.1\%$. We use the empirical Cox-Merz rule to relate the complex viscosity to the shear viscosity:

Set 1	Relaxation time λ_0 (ms)
1	N/A
2	N/A
3	1.2
4	1.5
5	N/A
6	0.8
7	0.7
8	3.0
Set 2	Relaxation time λ_0 (ms)
9	N/A
10	0.3
11	0.6
12	0.6
13	0.8
14	1.2
Set 3	Relaxation time λ_0 (ms)
15	N/A
16	2.0
17	2.0
18	7.0
19	5.5
20	5.2

Supplementary Table I. **Relaxation times of bouillons as obtained from pinching experiments.** We followed the evolution of the minimum neck diameter $D_{min}(t)$ in time for the three sets of solutions studied (see Table 1 in the main text). The relaxation times λ_0 of the solution are reported only when a characteristic exponential decay $D_{min}/D_0 = \exp(-t/(3\lambda_0))$ is observed; N/A is shown otherwise.

* A.Deblais@uva.nl
† Krassimir.Velikov@unilever.com

$$\eta(\dot{\gamma})\Big|_{\dot{\gamma}=\omega} = |\eta^*(\omega)| \quad (1)$$

In addition, we calculate the complex viscosity from the small amplitude oscillatory shear (SAOS) data:

$$|\eta^*(\omega)| = \frac{\sqrt{G'(\omega)^2 + G''(\omega)^2}}{\omega}. \quad (2)$$

This is reported in Supplementary Fig. 2, where we compare these results to the shear viscosity obtained from steady state flow (see Figure 1C, main text). Both agree very well. This is not surprising considering the very weak viscoelasticity of our samples as reported in [1]. Here, we do not need to invoke any correction parameters to collapse the data on the same master curve.

Supplementary Note 3: Parameter α in the dynamic squeezing model

In deriving the stress generated by a dynamic squeezing flow, we made the assumption that $\alpha = \frac{hn}{n+1}\sqrt[n]{\frac{h}{\kappa}\frac{\partial P}{\partial x}} \ll V$. Here, we provide more information about this assumption. To study the evolution of α with time, we first evaluate the term $\frac{\partial P}{\partial x}$. To this end, we start by noting that the pressure within the dynamics squeezing flow has the shape of a parabola, from line 341 (main text):

$$P(x, y) = C(R^2 - x^2 - y^2) \quad (3)$$

To calculate C, we use the fact that the integral over the pressure yields the normal force F_N:

$$F_N = \iint_{(x,y)} P(x, y)\, dx\, dy \quad (4)$$

We calculate this in cylindrical coordinates, where we have $x^2 + y^2 = r^2$:

$$F_N = 2\pi C \int_{r=0}^{r=R} (R^2 - r^2) r\, dr = \frac{\pi R^4 C}{2} \quad (5)$$

This implies that:

$$C = \frac{2F_N}{\pi R^4} \quad (6)$$

We combine this with Eq. 3 to find the value of $\frac{\partial P}{\partial x}$:

$$\frac{\partial P}{\partial x} = -2Cx = \frac{4F_N x}{\pi R^4} \quad (7)$$

Since we want to test whether α is smaller than V, we should maximize $\frac{\partial P}{\partial x}$. To this end, we evaluate this term at the position $x_{\max} = -R$:

$$\frac{\partial P}{\partial x}\Big|_{x_{\max}} = \frac{4F_N}{\pi R^3} = 4.1 \times 10^4\ \mathrm{Pa\ m^{-1}}, \quad (8)$$

where we have used that $F_N = 0.5$ N and $R = 2.5$ cm.

This now allows us to compute α as a function of time for different samples. Supplementary Fig. 3 shows $\alpha(t)$ for samples 1, 4, 8, and 14 (numbering referring to Table 1 in the main text). We conclude that the approximation $\alpha \ll V$ holds for most of the flow process, except for $t < 0.2$ s. As in our model of the process of eating bouillon, the stress generated by the flow is evaluated at $t = 1.2$ s, this condition is fully satisfied, independent of the bouillon composition.

Supplementary Note 4: Tongue velocity

The tongue velocity V has been studied quite intensively and reported [4]. For the low-viscous product as we consider in our study, the longitudinal velocity of the tongue reach 15 cm/s. We checked the effect of three different tongue velocities (10,15 & 20 cm/s) on Eq. 4 in the main text, as shown in Supplementary Fig. 4. The higher the velocity, the higher stress is generated. For all the velocities, a logarithmic law (Weber-Fechner) fits well the data and we show the corresponding goodness of fit -adjusted R^2- in the inset of Supplementary Fig. 4.

Supplementary Note 5: Statistical analysis of the models

It is difficult to compare the fits of mouthfeel thickness versus calculated stress data by a logarithmic and power law relation only based on the results of set 1 and 2, as the range of stresses investigated for the liquid bouillons is inherently limited. When comparing the residual sum of squares (RSS) given by the two different fits (22 vs. 25), no conclusion can be drawn. This is also the case when doing an F-test on the two residual populations given by the two different fits (Fig. 3B, main text). Since the p-value is superior to the significance level (chosen to be 5%), the null hypothesis (variances are equals) is accepted. The sample standard deviations of the residual populations are almost equal (0.27 vs 0.28). In other words, the difference between the sample standard deviations of the two populations is not statistically significant: we cannot prefer one fit over another only based on our liquid bouillons. We nevertheless stress that the power law model is not comparable to the logarithmic fit since an extra free parameter is added here, and that in the case of the logarithmic fit, we fully conserve the functional form. From an equivalent sum of residuals, one

would always prefer the simplest model. When adding the data of set 3 that enlarge the range of stress over two decades, we confirm that the logarithmic relationship is the best choice.

Supplementary Note 6: Determination of the constant c_1 in Eq. (9)

We use coordinates with $z \in [\,o, h]$ for the determination of the constant c_1; choosing $z \in [-\frac{h}{2}, \frac{h}{2}]$ does not change the result:

$$u_x(z) = \frac{hn}{n+1}\sqrt[n]{\frac{h}{m}\frac{\partial P}{\partial r}}[(c_1 + \frac{z}{h})^{\frac{(n+1)}{n}} + c_2], \tag{9}$$

with

$$\alpha \equiv \frac{hn}{n+1}\sqrt[n]{\frac{h}{m}\frac{\partial P}{\partial r}} \tag{10}$$

and

$$k \equiv \frac{(n+1)}{n} \tag{11}$$

, we have:

$$u_x(z) = \alpha[\,(c_1 + \frac{z}{h})^k + c_2] \tag{12}$$

The boundary condition $u_x(z=0) = 0$ gives:

$$c_2 = -c_1^k \tag{13}$$

which yields

$$u_x(z) = \alpha[(c_1 + \frac{z}{h})^k - {c_1}^k] \tag{14}$$

The boundary condition $u_x(z=h) = V$ gives:

$$V = \alpha[(c_1+1)^k - c_1^k] \tag{15}$$

that we solve for c_1. Because $\frac{V}{\alpha} \gg 1$, we have $c_1 \gg 1$. Expanding $(c_1+1)^k$ for large c_1 gives:

$$(c_1+1)^k \approx c_1 + kc_1^{(k-1)} \tag{16}$$

That leads to

$$\frac{V}{\alpha} \approx c_1^k + kc_1^{(k-1)} - c_1^k \approx kc_1^{(k-1)} \tag{17}$$

Substituting $k \equiv (n+1)/n$ back:

$$\frac{V}{\alpha} = \frac{n+1}{n}c_1^{\frac{1}{n}} \tag{18}$$

Leading to the result shown in Eq. 9 in the manuscript:

$$c_1 = (\frac{n}{n+1})^n \frac{V^n}{\alpha^n} \tag{19}$$

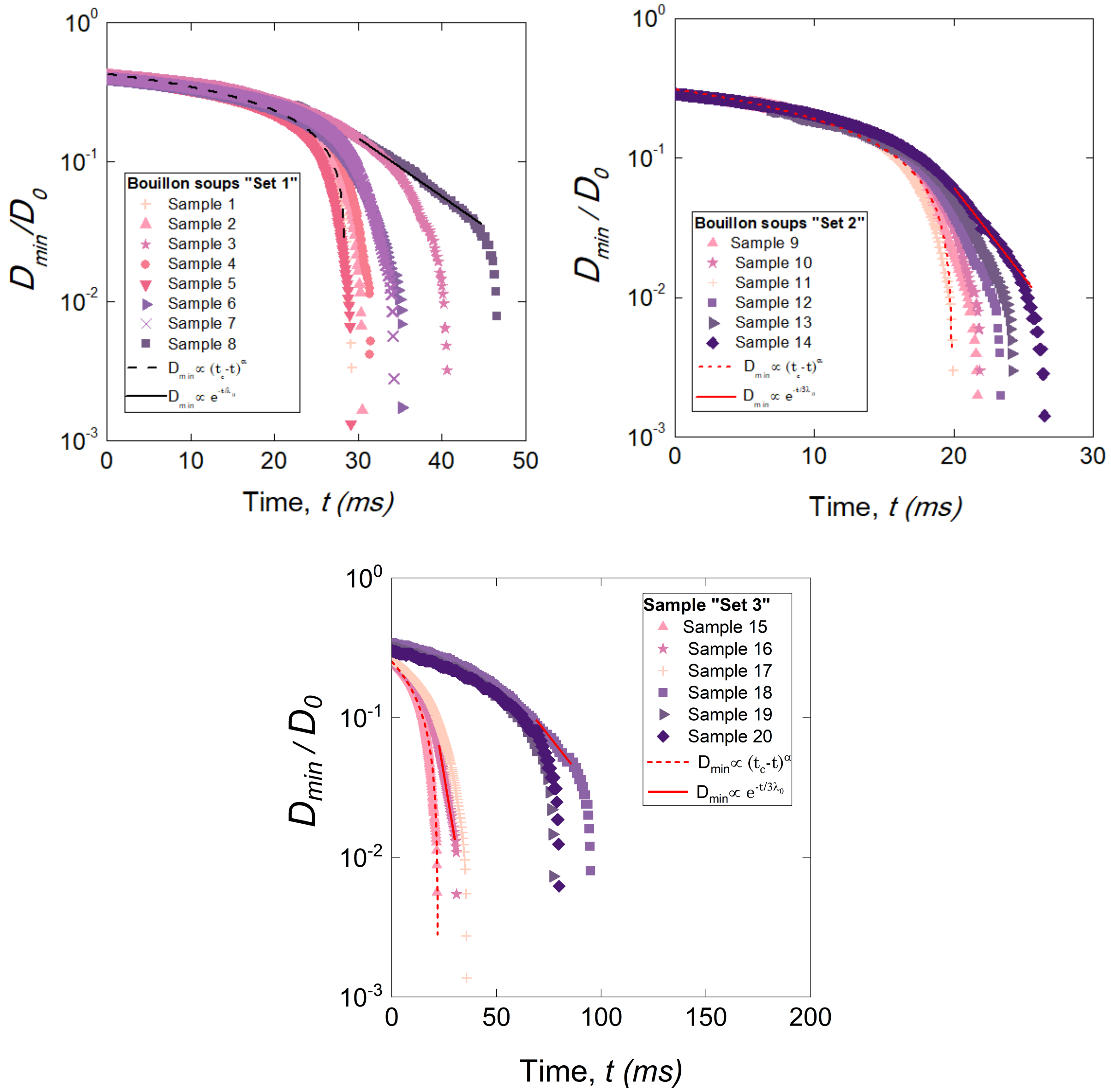

Supplementary Figure 1. **Thinning dynamics as obtained from filament rheometry measurements on all samples at 40°C.** Solid lines indicate exponential regimes $D_{\min}/D_0 = \exp(-t/3\lambda_0)$ characteristic of a viscoelastic behavior. The relaxation times λ_0 extracted from these dynamics are reported in the Supplementary Table . Dashed lines are power law fits $D_{\min}/D_0 \propto (t_c - t)^{\alpha}$.

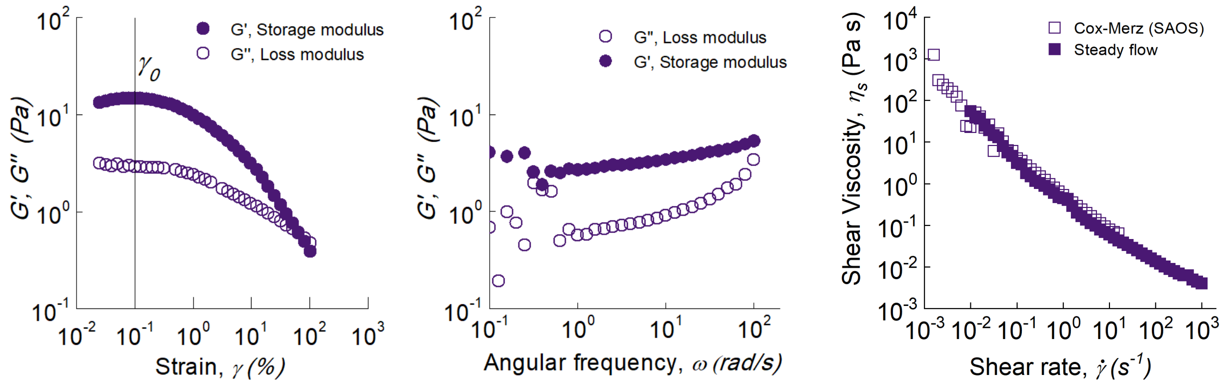

Supplementary Figure 2. **Rheology measurements of Sample 14.** (Left) Storage and loss module (G' and G") as a function of strain; these strain sweep experiments indicate that the linear viscoelastic regime extends to $\gamma_0 < 0.1\%$. (Middle) Frequency sweeps of G' and G" within the viscoeleastic regime, from which we can calculate the complex viscosity from eq.2. (Right) Both collapse on the same master curve, in agreement with the Cox-Merz rule (eq. 1.)

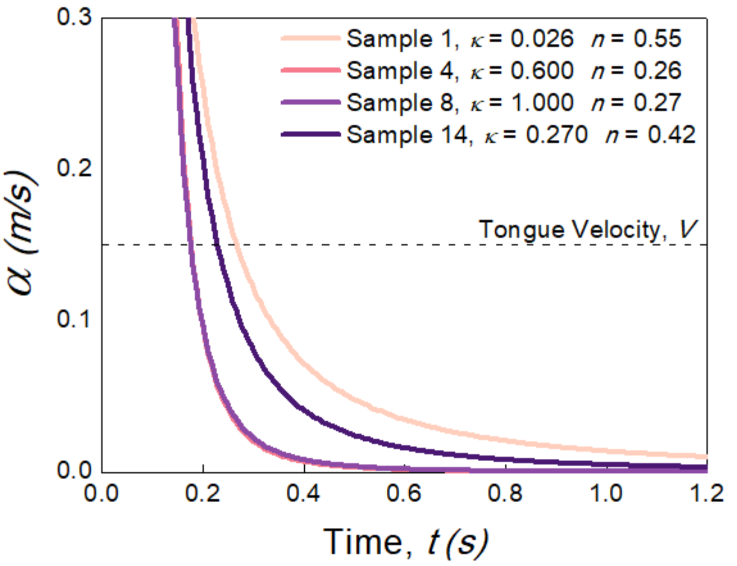

Supplementary Figure 3. **Model parameter α as function of time for four samples considered in this study** (varying in composition and in rheological parameters κ and n). The dashed line indicates the tongue velocity assumed in our experiments.

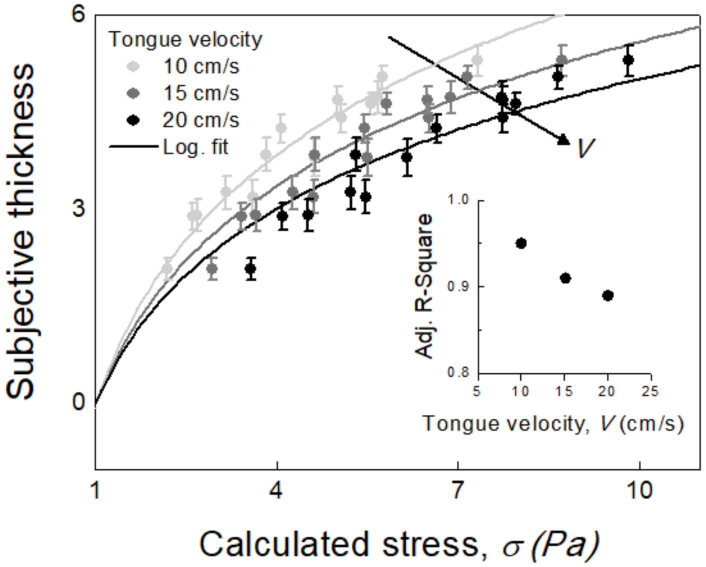

Supplementary Figure 4. **Effect of tongue velocity V on the calculated stress σ.** Three different velocities are computed (10, 15, and 20 cm/s) and show that the higher the velocity, the higher the calculated stress. The Adjustment R-square (inset) is still good (>0.9) for these velocities and decreases with velocity. The tongue velocity for the low-viscous product is 15 cm/s [4].

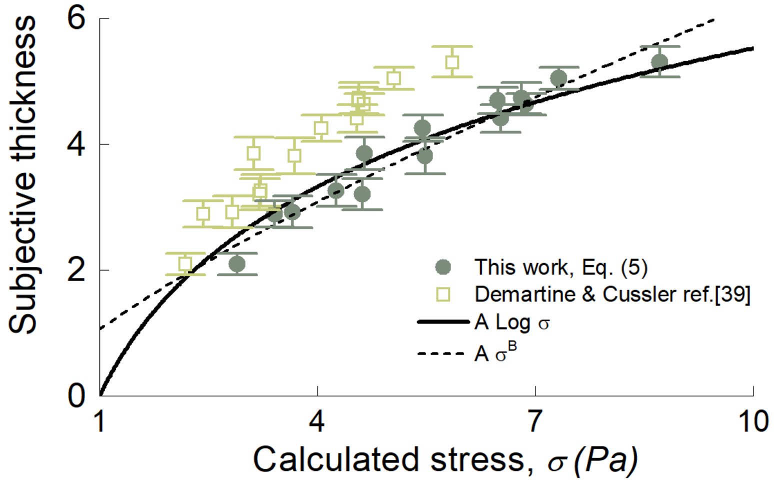

Supplementary Figure 5. **Generated stress σ given by Eq .5 (main text, filled circles) compared to the static squeezing model initially derived by Demartine in [5], (empty squares). The generated stress is underestimated in the latter case, and the sample's rheology (*i.e.*, consistency κ index and power law n) have a non-linear effect on σ.**

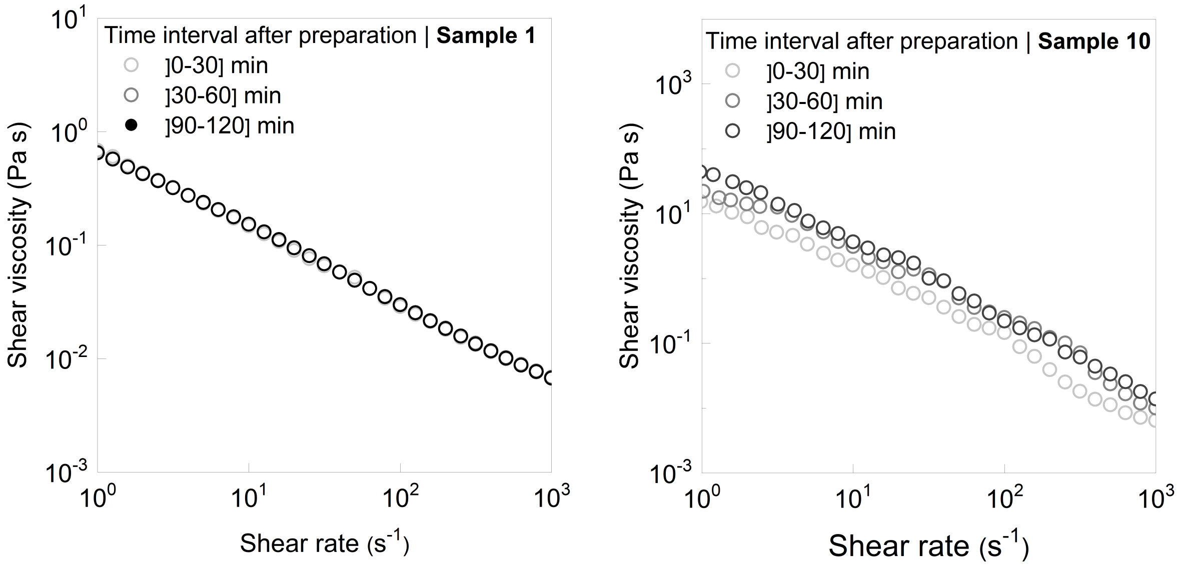

Supplementary Figure 6. **Effect of waiting time on the rheology of the sample after preparation.** At low concentration of thickeners (left), the rheology of the liquid sample remains unchanged in the interval time studied. At higher concentrations (right), the shear viscosity can slightly increase because of the slow swelling of the thickeners (starches and xanthan gum). All of the rheology measurements and sensory analysis have been performed immediately after preparation, in the time interval [0 − 30] min for the bouillon samples (set 1 and 2).

Supplementary Table II. **List of attributes used to describe the different sensory properties of the set 1 & 2. For set 3, see ref. [6].**

–Appearance–	
**Attribute**	**Definition**
Yellow	Yellow intensity
Brown	Brown intensity
Amount of fat	Surface covering with fat
Size of fat	Size of fat lumps floating on the bouillon
Viscosity	Degree of viscosity (flowing speed from the spoon)
Turbidity	Degree of turbidity
Amount of vegetable particles	Amount of particles coming from vegetables (carrot, celery, etc.)
Amount of herb particles	Amount of particles coming from herbs (parsley, chives, etc.)
Amount of spice/speckles	Amount of small speckles (coming from spices such as pepper)
Residual layer on spoon	Degree of layer left behind on metal spoon
**–Odor–**	
**Attribute**	**Definition**
intensity	Odor intensity, overall odor
**–Taste–**	
**Attribute**	**Definition**
Intensity	Taste intensity
Salt	Degree of salty taste
Sour	Degree of sour taste
Sweet	Degree of sweet taste
Bitter	Degree of bitter taste
Umami	Degree of umami in the taste
Savory	Degree of savory like flavor
Meat	Degree of overall meat flavor (total flavor of poultry, beef etc)
Chicken	Degree of overall chicken flavor
Beef	Degree of overall beef flavor
Roasted	Degree of roasted meat flavor
Vegetables	Degree of vegetable flavor
Herbs	Degree of herb flavor
Spices	Degree of spice flavor (nutmeg, pepper, curry etc.)
Starch/noodles	Degree of starchy/noodle flavor
Balance	Degree of harmony in the flavor
Complexity	Degree of complexity of the flavors, amount of separate flavors
**–Mouthfeel–**	
**Attribute**	**Definition**
Thick	Thickness of product while moving it in the mouth
Fat	Degree of fatty mouthfeel
Full	Degree of full, umami-like, mouthfeel
Astringent	Degree of astringent feeling on the tongue and cheeks

[1] C. E. Wagner, A. C. Barbati, J. Engmann, A. S. Burbidge, and G. H. McKinley, Quantifying the consistency and rheology of liquid foods using fractional calculus, Food Hydrocolloids **69**, 242 (2017).
[2] C. Clasen, J. Plog, W.-M. Kulicke, M. Owens, C. Macosko, L. Scriven, M. Verani, and G. H. McKinley, How dilute are dilute solutions in extensional flows?, Journal of Rheology **50**, 849 (2006).
[3] G. Brindley, J. Davies, and K. Walters, Elastico-viscous squeeze films. part i, Journal of Non-Newtonian Fluid Mechanics **1**, 19 (1976).
[4] T. H. Shawker, B. Sonies, M. Stone, and B. J. Baum, Real-time ultrasound visualization of tongue movement during swallowing, Journal of Clinical Ultrasound **11**, 485 (1983).
[5] M. L. Demartine and E. L. Cussler, Predicting subjective spreadability, viscosity, and stickiness, Journal of Pharmaceutical Sciences **64**, 976 (1975).
[6] A. E. Blok, D. P. Bolhuis, H. V. Kibbelaar, D. Bonn, K. P. Velikov, and M. Stieger, Comparing rheological, tribological and sensory properties of microfibrillated cellulose dispersions and xanthan gum solutions, Food Hydrocolloids , 107052 (2021).
